# Tick Saliva and the Alpha-Gal Syndrome: Finding a Needle in a Haystack

**DOI:** 10.3389/fcimb.2021.680264

**Published:** 2021-07-20

**Authors:** Surendra Raj Sharma, Shahid Karim

**Affiliations:** Center for Molecular and Cellular Biology, School of Biological, Environmental, and Earth Sciences, University of Southern Mississippi, Hattiesburg, MS, United States

**Keywords:** tick, α-gal, alpha-gal syndrome, red meat allergy, hypersensitivity, sugar, microbiome

## Abstract

Ticks and tick-borne diseases are significant public health concerns. Bioactive molecules in tick saliva facilitate prolonged blood-feeding and transmission of tick-borne pathogens to the vertebrate host. Alpha-gal syndrome (AGS), a newly reported food allergy, is believed to be induced by saliva proteins decorated with a sugar molecule, the oligosaccharide galactose-⍺-1,3-galactose (α-gal). This syndrome is characterized by an IgE antibody-directed hypersensitivity against α-gal. The α-gal antigen was discovered in the salivary glands and saliva of various tick species including, the Lone Star tick (*Amblyomma americanum*). The underlying immune mechanisms linking tick bites with α-gal-specific IgE production are poorly understood and are crucial to identify and establish novel treatments for this disease. This article reviews the current understanding of AGS and its involvement with tick species.

## Introduction

Ticks are obligate ectoparasites of vertebrates and depend on hematophagy for nutrition at each stage of their life history. Because of their hematophagous behavior ticks serve as competent vectors of viruses, bacteria, and protozoan pathogens, and are thus important organisms from a global health perspective (Parola and Raoult, 2001). Hematophagy and host specificity of Ixodid ticks contribute to their ability to acquire, maintain, and transmit multiple pathogens and cause tick-bite-associated diseases, such as alpha-gal syndrome (AGS) and tick paralysis (Rochlin and Toledo, 2020). During blood feeding on their host, ticks secrete and introduce a plethora of salivary secretions that modulate the host immune responses and inoculate tick-borne pathogens (Jongejan and Uilenberg, 2004). Several of these pathogens are believed to be responsible for tick-borne infections such as, viral diseases (e.g., Tick-borne encephalitis, Powassan encephalitis, Colorado tick fever, and Omsk hemorrhagic fever), protozoan disease (e.g., babesiosis and theileriosis), and bacterial diseases (e.g., Lyme disease, Rocky Mountain spotted fever, Anaplasmosis, Rickettsiosis, Ehrlichiosis, and Tularemia) (Schwan and Piesman, 2002; Socolovschi et al., 2009; Brites-Neto et al., 2015; Chmelař et al., 2016; Rochlin and Toledo, 2020). In the United States alone, a surveillance study conducted by the Centers for Disease Control and Prevention (CDC) in the period of 2004–2016 reported that, 77% of vector-borne disease cases are caused by ticks (Rosenberg et al., 2018). Of the several diseases vectored by ticks, Lyme disease is the most prevalent across the northern hemisphere. The CDC estimates that approximately 476,000 people are diagnosed with the Lyme disease each year in the United States (Schwartz et al., 2021). The economic burden caused by tick-borne diseases is increasing each year, and the annual cost of Lyme disease to the United States health care system ranges between $712 and $1.3 billion, or approximately $3,000 per patient (Adrion et al., 2015). In recent years, several tick species are moving and expanding their geographic range. Hence, studies have predicted an increase in tick-borne diseases, including AGS (Commins et al., 2009; Monzón et al., 2016; Raghavan et al., 2019).

Food allergies affect ~32 million Americans, including 5.6 million children under 18 years of age (Facts and Statistics, 2019). More than 170 types of food can cause allergies, including milk, eggs, peanuts, tree nuts, wheat, soy, red meat, fish, and crustacean shellfish (Commins, 2015; Iweala et al., 2017; Iweala et al., 2018; Yu et al., 2016). Food allergies are responsible for many severe allergic reactions in the United States, and AGS is already common in several regions of the world. In the US alone, the number of confirmed cases of AGS has risen from only 12 in 2009 to 34,000 in 2019 (Wilson and Platts-Mill, 2019; Alphagalinformation.org, 2020; Commins, 2020; Plats-Mill et al., 2020; Binder et al., 2021). Commins (2020) predicted that the percentage of individuals living in endemic tick areas that have been sensitized to α-gal ranges from 15–30%. Furthermore, this syndrome is the leading cause of the onset of allergy and anaphylaxis in adults in the United States and is prevalent in the southeastern United States (Pattanaik et al., 2018; Binder et al., 2021; Alpha-Gal Syndrome Subcommittee Report to the TBDWG, 2020). Clinical manifestations of AGS vary among patients, and the onset of AGS may not show clinical signs in sensitized patients. However, α-gal sensitization has been reported as a significant risk factor for coronary heart disease, even in people lacking clinical symptoms (Wilson et al., 2019). This review focuses on our current understanding of ticks, including their sialomes, intrinsic factors, and associations with the onset of AGS.

## Alpha-Gal Syndrome: A Paradigm-Shifting Allergy

Galactose-α-1,3-galactose (α-gal) is a disaccharide sugar found in mammalian glycolipids and glycoproteins, except in Old World monkeys, apes, and humans (Galili and Avila, 1999; Galili, 2001; Galili, 2005; Galili, 2013a; Apostolovic et al., 2014; Hilger et al., 2016; Iweala et al., 2017; Iweala et al., 2020). Alpha-gal has also been reported in bacteria, protozoa, fungi, and red algae (Galili and Avila, 1999; Hodzic et al., 2016; Khoury et al., 2018). In addition, many human pathogens and viruses attach α-gal to glycoproteins (Galili and Avila, 1999; Galili, 2013a; Galili, 2020). Generally, non-mammalian vertebrates lack expression of α-gal, but with a few exceptions, such as cobra venom, teleost fish eggs, and amphibian skin (Galili and Avila, 1999; Galili, 2001; Gowda et al., 2001). Unlike protein antigens, α-gal is a unique antigen that is not denatured by high cooking temperatures, and it is one of the two carbohydrates associated with life-threatening allergic reactions (Apostolovic et al., 2014; Soh et al., 2015). Hilger et al. (2016) reported that proteins responsible for red meat allergic reactions are glycosylated with α-gal. Takahashi et al. (2014) also analyzed α-gal antigens in beef and identified new transmembrane proteins, which were aminopeptidase N (AP-N) and angiotensin-converting enzyme 1 (ACE-1). Furthermore, several other heat-stable antigens present in red meat i.e., α and β enolase, amino transferase, and creatinine kinase, are also reported to be cross-reacting with red meat allergy patient serum as well as with anti-α-gal antibodies (Apostolovic et al., 2014).

AGS, also known as mammalian meat allergy, red meat allergy, or idiopathic allergy, is a unique type of allergy that involves an IgE antibody response to α-gal in humans (Commins et al., 2009). Since its discovery in 2007, several efforts have been made to understand this novel form of food allergy (Commins et al., 2011). Typically, food allergies are classified into 1) IgE-mediated or 2) cell-mediated (also known as non-IgE-mediated). The IgE-mediated allergic pathway demonstrates the rapid onset of clinical symptoms in less than 30 min after antigen exposure (Savage et al., 2016; Waserman et al., 2018). As a clinical hallmark, AGS α-gal reactions are often severe and sometimes fatal (Fischer et al., 2016). Moreover, depending on the antigen’s route, source, and nature, the onset of clinical symptoms of AGS can be immediate or delayed for 2–10 h (Commins et al., 2009; Commins et al., 2016; Steinke et al., 2015). Rapid onset of anaphylactic reactions was reported with cetuximab, a monoclonal antibody, in AGS patients (Chung et al., 2008).

However, as an idiosyncratic clinical feature (**Supplementary Figure 2**) of AGS, delayed reactions are reported in patients after red meat consumption (Platts-Mills et al., 2015a; Wilson et al., 2017). The mechanism of delayed reaction against red meat in AGS patients is poorly understood; however, it is correlated with several factors involved in meat digestion, absorption, transport, and subsequent presentation to the host immune system (Steinke et al., 2015; Platts-Mills et al., 2015b). An alteration of lipid metabolism is also the main contributor to the delayed response due to the delayed appearance of α-gal-associated glycolipids (Steinke et al., 2016; Iweala et al., 2017). Age and atopy are also reported as the cause of AGS development (Gonzalez-Quintela et al., 2014; Villalta et al., 2016; Fischer et al., 2017). In general, AGS occurs in people of all ages with no known genetic predisposition (Commins et al., 2012; Wilson and Platis-Mills, 2019). AGS patients exhibit various clinical symptoms, including urticaria, angioedema, pruritus, and systematic anaphylaxis. Some patients have reported specific symptoms, such as nausea, indigestion, diarrhea, and abdominal discomfort, before AGS onset. However, even after exposure to α-gal, other patients reported no appearance of the symptoms listed above, which further highlights the unusual nature of AGS (Platts-Mills et al., 2015b; Wilson et al., 2017). The reported diverse clinical manifestations in AGS patients might be related to the nature of the allergen and dose as well as the presence of other cofactors, such as metabolic variations (Morisset et al., 2012; Wölbbing et al., 2013; Fischer et al., 2016). Variations of lipid or fatty acid metabolism in the host delays the appearance of α-gal in the bloodstream and AGS symptom development (Steinke et al., 2016). Similarly, the allergen dose and associated host cofactors play a critical role in the progression and severity of AGS (Morisset et al., 2012; Fischer et al., 2016; Wilson et al., 2018; Wilson et al., 2019). An elegant study compared the frequency of delayed anaphylactic reactions against α-gal in AGS patients subjected to beef, pork, lamb, and deer meat. Interestingly, the frequency of delayed anaphylactic responses was 53, 47, 9.1, and 7.3%, respectively (Fischer et al., 2016). One reason for such variation in delayed anaphylactic reactions against red meats in AGS patients might be the presence of variable quantities of the α-gal epitope and adjuvant factors, such as lipids (Hendricks et al., 1990; Fischer et al., 2016). The biochemical composition of red meat, its processing, ingestion, and absorption are all equally important in the onset of anaphylactic reactions in AGS patients (Commins et al., 2016). Wölbing et al. (2013) used an oral challenge approach to study the role of cofactors associated with red meat. Including this study, few other studies identified various exogenous and endogenous factors, such as alcohol, physical exercise, non-steroid analgesic drugs, and menstruation, to be vital in proliferation or increasing severity of the reaction against the red meat (Wölbing et al., 2013; Fischer et al., 2014; Versluis et al., 2016). In addition to red meat, several other food products and medicines containing the α-gal antigen, such as gelatin, collagen, and cetuximab, can also cause AGS (Commins et al., 2016; Commins, 2020). Therefore, the use of drugs derived from mammalian products also poses a risk to AGS patients and exacerbates allergic reactions (Commins et al., 2016). Additionally, a high titer of IgE antibodies to α-gal in AGS patients adds several complications for cardiovascular disease patients (Wilson et al., 2018). The development of AGS has been a complex mystery. Indeed, some studies have reported an association between tick bites and AGS, the mechanistic details of how a tick bite can lead to the priming of immune cells during hematophagy are still unclear (Commins et al., 2009; Commins et al., 2016; van Nunen, 2018).

## A Single Sugar Makes All the Difference: The Significance of α-gal

All mammals have an α-1,3-galactosyltransferase (α1,3GT) enzyme encoded by the *GGTA1* gene; however, during the evolution of Old-World monkeys, apes, and humans, this gene was inactivated due to a frameshift mutation (Galili, 2015). The α1,3GT enzyme is responsible for generating α-gal by transferring a galactose residue with an α-1,3 linkage to the terminal lactosaminide (Gal-β-1,4-GlcNAc-R) on glycolipids and glycoproteins (Galili, 2001; Macher and Galili, 2008). Interestingly, the *GGTA1* gene appears to have become nonfunctional during mammalian evolution; while it is still active in marsupials (Lantéri et al., 2002). Since this gene is in the form of a pseudogene in humans, it does not express α-gal epitopes. Therefore, humans develop antibodies against α-gal, which gives them an advantage in fighting against α-gal expressing pathogens (Welsh et al., 1998; Galili, 2013b). Several studies have discussed the benefit of anti-α-gal antibody development in humans, including immunogenic stimulation against parasites expressing α-gal epitopes, such as *Trypanosoma* and *Leishmania* species (Avila et al., 1989). A study conducted in the α-1,3GT-knockout (*GGTA1*-Ko or α1,3GT^KO^) mouse model by raising anti-α-gal antibodies demonstrated that they induce anti-α-gal IgG and IgM antibodies upon inoculation with the human pathogen *E. coli* O86:B7 (Posekany et al., 2002; Yilmaz et al., 2014). Another study demonstrated a decrease in malarial parasite transmission due to the high titer of anti-α-gal IgM antibodies (Yilmaz et al., 2014). These discoveries sparked an interest in α-gal pan-vaccines; that is, vaccinating against pathogens or vectors expressing α-gal to prevent infections (Soares and Yilmaz, 2016). Two independent studies using this approach successfully reduced *Leishmania* infections in a α-1,3GT^KO^ mouse model (Iniguez et al., 2017; Moura et al., 2017). IgG antibodies against α-gal are highly abundant and are estimated to be present in the 30–100-μg/ml range in human serum (Galili et al., 1984; Galili et al., 1993). Anti-α-gal IgG antibodies persist in newborns at a low level for up to 6 months and gradually increase over 2–4 years until they reach their highest level, which is equivalent to the levels in adults (Galili et al., 1993). The definitive cause, source, and nature of the antigens involved in rising α-gal antibody levels at early ages are yet to be determined. Alpha-gal is expressed by various microbes, including *Escherichia*, *Klebsiella*, and *Salmonella* and many of these bacteria belong to human gut microbiome hence production of anti-α-gal antibody may be one way to withstand microbial proliferation or confer protection from detrimental effects of pathogen colonization in human body (Galili et al., 1988; Galili, 2013b; Shreiner et al., 2015).

## Alpha-Gal Syndrome: An Emerging Worldwide Phenomenon

This emerging tick bite induced food allergy has been reported to occur in seventeen nations worldwide (**Table 1** and **Supplementary Figure 1**). The discovery of AGS worldwide has opened a new avenue for making a connection between AGS patients and tick bites. It has provided insight into how bites from different tick species can induce IgE sensitization in humans. In a few countries, AGS onset was linked to tick bites, however, a direct link between previous tick bites and AGS has not been established (Chinuki et al., 2016). Tick bites were first implicated in AGS in Australia, although α-gal was not presented as the cause (Steinke et al., 2015; van Nunen, 2015). Loh and Tang, (2018) reported that Australia is among the countries with the highest AGS and anaphylaxis rates in the world. Similarly, an earlier report estimated that the prevalence of AGS in tick endemic regions is one in every 550 people and is predicted to surge (van Nunen, 2015). In Australia, including the south coast of New South Wales and Sydney coast, AGS cases coinciding with the endemic area inhabited by the *Ixodes holocyclus* tick have been reported (van Nunen, 2014; Steinke et al., 2015; van Nunen, 2015; van Nunen, 2018). There is an interesting story related to discovering the association between tick bite and red meat allergy in the United States. In 2008, in a clinical trial of the monoclonal antibody cetuximab, cancer patients induced IgE antibodies to α-gal (Chung et al., 2008). In the same year, an increasing trend in a number of patients with delayed-type red meat allergy were reported in the southeastern United States. A surveillance study conducted by the CDC from 2012–2013 showed significantly higher α-gal-directed IgE levels in the southeastern United States, an established *A. americanum* tick population territory (Tick and Mammalian Meat allergy, 2021). Furthermore, a link between the tick and α-gal-related hypersensitivity became more evident when the same surveillance study reported the overlapping of IgE prevalence and the geographical distribution of *A. americanum*. Commins et al. (2011) reported a direct link between tick bites and the development of IgE antibodies to red meat, which further supported the hypothesis that *A. americanum* tick bites are associated with AGS onset. The incidence of AGS is increasing in the southwest and eastern coastal regions of the United States, which correlates with the expansion and distribution of the Lone Star tick (Commins et al., 2009; Steinke et al., 2015; Raghavan et al., 2019; Commins, 2020). In the United States, the first reports of AGS in 2009 included only 24 officially reported cases, but a recent study put the number at 34,000 confirmed cases (Binder et al., 2021). Wilson et al. (2018) reported an 32% increase of AGS cases in the southeastern United states, where the Lone Star tick is prevalent. Public repositories show that up to 3% of the population has AGS (Alpha-gal info, 2020), while misdiagnosed or undiagnosed cases cannot be ruled out (Commins, 2020). The reported AGS cases in Japan suggest that a tick species is responsible for the allergy (Takahashi et al., 2014). Based on the presence of α-gal in its salivary glands, the *Haemaphysalis longicornis* tick has been suggested to be causing AGS (Chinuki et al., 2016). AGS cases in Korea are also believed to be associated with *H. longicornis* tick bites (Chinuki et al., 2016). Similarly, Hamsten et al. (2013) identified traces of α-gal in the mid-gut of the *Ixodes ricinus* tick, which led to the belief that it was involved in causing red meat allergy in Sweden. In this study, researchers compared α-gal epitopes from *A. americanum* and *I. ricinus* ticks and reported that they share certain characteristics, although there were specific variations. Additionally, other countries with reported AGS cases include Spain, Germany, Turkey, and Switzerland (van Nunen, 2015). Interestingly, numerous African countries that conducted seroprevalence studies found that individuals have IgE antibodies specific to α-gal. However, there was no indication of any allergic reactions after red meat consumption (Commins and Platts-Mills, 2013a). This observation has led to questions regarding the actual cause of α-gal-specific IgE production in those individuals, and it was hypothesized that the cause could include cestodes, ticks, and other ectoparasites (Commins and Platts-Mills, 2013b). A small number of AGS cases in a rural farming community in South Africa suggested a need to conduct more in-depth studies (van Nunen, 2015). The patients with AGS recalled having a tick bite before the onset of AGS symptoms, although the tick species has yet to be determined. Information about AGS cases across Central America is not available. However, Araujo et al. (2016) reported that injected saliva or bites from the tick species belonging to *Amblyomma cajennese* complex. *Amblyomma sculptum* induced specific IgE antibodies in an α-1,3-GT^KO^ mouse. Similarly, the tick species belonging to *A. cajennese* complex, prevalent in Costa Rica and neighboring countries, are thought to be involved in causing AGS (Wickner and Commins, 2014). Several other tick species found in various South and Central American regions belonging to the *Amblyomma* and *Ixodes* genera are known for biting humans, although a link to AGS has not yet been established (van Nunen, 2015).

**Table 1:** List of tick species reported to be associated with alpha-gal syndrome worldwide.

Associated Ticks#	Country	Reference
*Amblyomma americanum*	USA	Crispell et al. (2019); Platts-Mill and Commins, (2013); Khoury et al. (2018)
*Ixodes holocyclus*	Australia	Hamsten et al. (2013); Apostolovic et al. (2014)
*Ixodes australiensis*		
*Ixodes ricinus*	Sweden	Hamsten et al. (2013); Gray et al. (2016); Bircher et al. (2017); Schmidle et al. (2019); van Nunen (2018); Mullins et al. (2012); Caponetto et al. (2013); Jappe (2014); Calamari et al. (2015); Uasuf et al. (2018); Lied (2017)
	Switzerland	
	Italy	
	Germany	
	Norway	
*Rhipicephalus bursa*	Spain	Mateos-Hernández et al. (2017)
*Haemaphysalis longicornis*	Korea	van Nunen (2018); Lee et al. (2013); Sim et al. (2017)
	Japan	
*Amblyomma cajennese* species complex*.?*	Costa Rica	van Nunen (2015); van Nunen (2018)
	Panama	
*Not identified*	Zimbabwe	van Nunen (2015)
*Amblyomma testudinarium*	Japan	van Nunen (2018); Sekiya et al. (2012); Chinuki et al. (2016); Hashizume et al. (2018)
*Amblyomma sculptum*	Brazil	van Nunen (2018); Kaloga et al. (2016)
*Amblyomma cajennese* species complex*.s.s?*	Ivory Coast	
*Amblyomma variegatum*		
*Amblyomma herbraeum*	South Africa	van Nunen (2015); Gray et al. (2016)
Not identified	France	Jacquenet et al. (2009); Morisset et al. (2012); van Nunen (2015); Guillier et al. (2015); Keleş & Gündüz (2019); Berends and Elberink (2017)
	Turkey	
	Netherlands	

# Association between tick species and AGS is not experimentally established in the listed reports.?: Exact species variant is not specified in reports.

## The Origin of α-gal in Tick Saliva

In recent years, studies were primarily focused on identification and profiling of α-gal antigens in tick saliva and tissues to decipher the connection of a tick bite and AGS.

It is still unclear how tick acquires and presents α-gal and primes the host to develop immune response to develop anti-α-gal IgE. There are several possibilities these α-gal antigens may be residual or recycled mammalian glycoproteins or glycolipids from previous blood meal or may be α-gal signatures contributed by tick-acquired viruses, protozoans, or bacteria. However, various evidence suggests that α-gal is possibly originating from tick itself. Several studies reported presence of proteins in tick salivary gland, midgut, and saliva cross-reacting with serum from AGS patients and anti α-gal antibodies. Presence of α-gal was first reported in the midgut of *I. ricinus* (Hamsten et al., 2013). Araujo et al. (2016) also reported presence of α-gal antigen in *A. sculptum* saliva as well further provided evidence that injection of saliva derived α-gal antigen or feeding of ticks on α-1,3 GT^KO^ mice induce anti α-Gal IgE antibodies. Similarly, another study reported α-gal epitope-containing tick proteins in *Rhipicephalus microplus* BME/CTVM23 cells and in *Hyalomma marginatum* salivary glands (Mateos-Hernández et al., 2017). Furthermore, presence of several proteins from various groups namely vitellogenins, serpin, actin, α-macroglobulin, chitinase like lectin and transport or channel-forming proteins with α-gal epitope were also identified in protein extracts of *I. ricinus* larvae and adults (Apostolovic et al., 2020). Recently, the presence of α-gal-associated antigens in *A. americanum and I. scapularis* ticks was discovered *via* multiple approaches, which included, mass spectrometry, immunoblotting, and immunolocalization analysis of tick tissues (Crispell et al., 2019). This study also demonstrated that expression of α-gal antigens is highest in partially blood-fed *A. americanum* salivary glands and saliva. Furthermore, this study also provided evidence that these α-gal antigens are localized in salivary secretory vesicles (exosomes) of partially engorged *A. americanum* and *I. scapularis* ticks (Crispell et al., 2019). Furthermore, detection of α-gal in *A. americanum* tick fed on human blood, which lacks α-gal, further indicate that alternative recycling mechanism or mechanism producing α-gal might exist in ticks. Immunoblot analysis of *A. americanum* salivary extracts containing α-gal antigen following treatment with PNGase F further demonstrated that α-gal is bound with protein in N-linked glycosylated form (Crispell et al., 2019). The role of tick β-1,4-galactosyltransferase (β-1,4-GT) in α-gal expression was reported in *I. scapularis via* heterologous gene expression and localization of α-gal in α-gal-negative cells (Cabezas-Cruz et al., 2018). However, it is not clear whether proteins glycosylated by β-1,4-GT can also sensitize host to develop anti α-gal antibody and also cross-react with protein containing Galactose-α-1,3-galactose epitope. Intriguingly, the key enzyme, α1,3-GT, which synthesizes α-gal, remains unidentified in tick genomes.

Few studies have reported that tick-borne bacteria such as *Anaplasma phagocytophilum* and *Borrelia burgdorferi *sensu lato express α-gal and increase α-gal signature in ticks, hence, the role of tick microbiome as one possible source of α-gal cannot be negated (Cabezas-Cruz et al., 2018; Vechtova et al., 2018; Hodzic et al., 2019). Furthermore, several studies have reported that a few bacteria from Enterboacteriaceae family such as *Salmonella*; *Pseudomonas*, *Staphylococcus* as well as from Rizobiaceae and Caulobacteriaceae family possess enzyme α1,3-GT enzyme which can decorate protein with α-gal (Hamadeh et al., 1996; Brown et al., 2013; Montassier et al., 2019; Vechtova et al., 2018). Since bacteria from the same family and group are also reported in tick salivary microbiome, it will be intresting to investigate the impact of presence of bacteria in a tick and its relation with α-gal signature of tick (Maldonado-Ruiz et al., 2021). Role of tick’s microbiome in causing or increasing tick’s ability to develop or present α-gal antigen is an emerging area of research. In context of the role of tick microbiome in sensitization of humans against α-gal during tick feeding, the dual-allergen-exposure hypothesis seems plausible which states that dual exposure of α-gal antigen along with addition of tick microbiome in tick–host interaction interface can cause sensitization of host against α-gal.

## Host and Tick Factors Contributing to AGS

**Figure 1:**
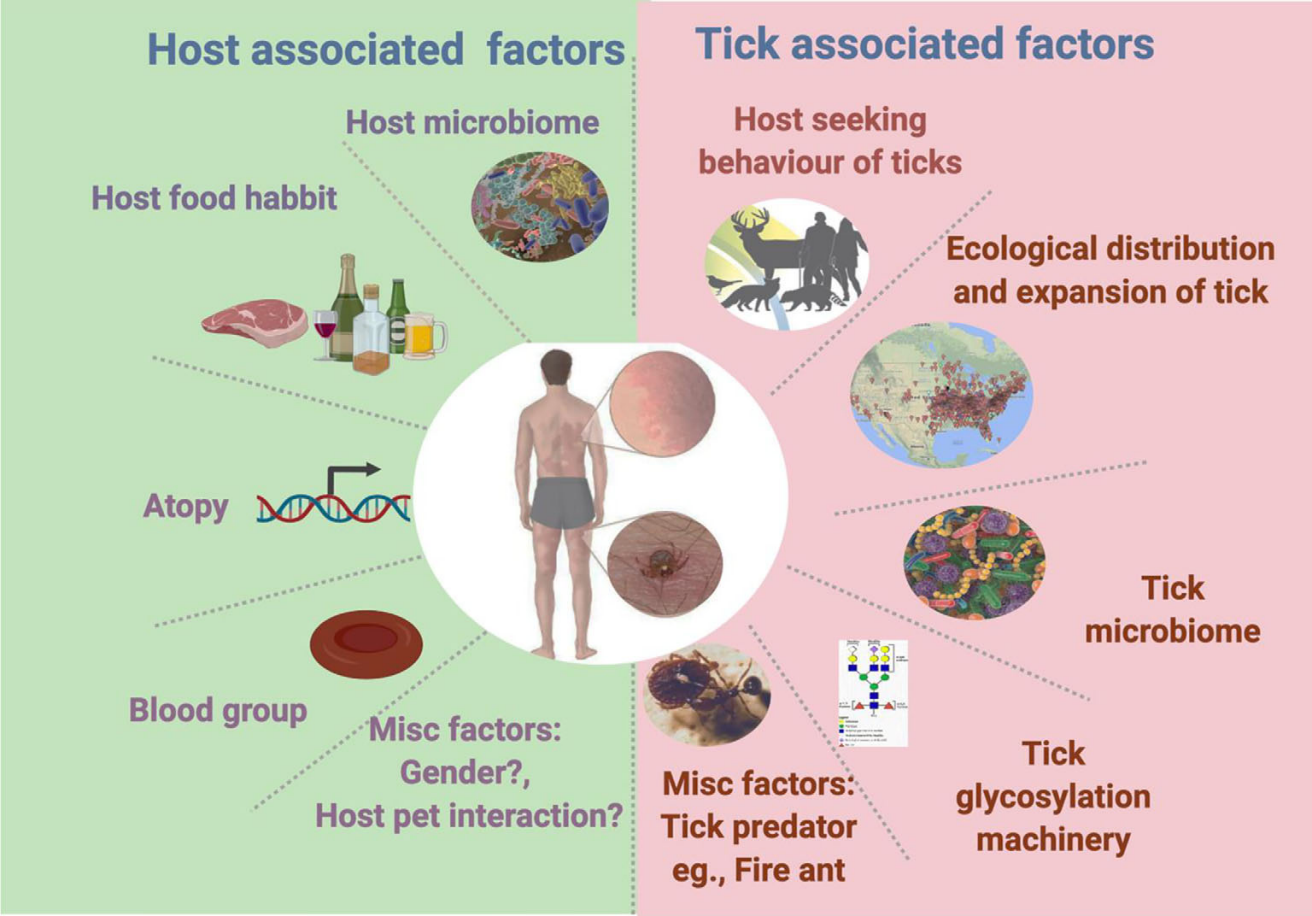
Tick- and host-associated factors linked with alpha-gal syndrome.

Information regarding host factors, which contribute towards development of AGS, is limited; however, there is a significant progress in this area. Studies have reported that despite presence of high titer of anti α-gal IgE, some people do not develop AGS (Michel et al., 2014; Villalta et al., 2016). Based on existing evidence few factors (**Figure 1**), which might contribute to such variation to the host, can be listed as a) host genetic factors such as blood group and atopy b) host microbiome and associated factors such as diet, and medication. Few research studies report variations in anti-α-gal response among people with different blood groups. In a study conducted in Sweden, people with blood type B negative were affected by the α-gal allergy more often than other blood types (Hamsten et al., 2013). However, this trend contradicts the hypothesis that a protective effect is produced by blood type B (Posthumus et al., 2010). Intriguingly, this relationship seems to be related to the similarities between α-gal and blood type B antigen (Gal-α1,3 (Fuc-α1,2)-Gal) structures (Posthumus et al., 2010; Bircher et al., 2017). Hamsten et al. (2013) examined the allergy incidence rates, whereas Posthumus et al. (2010) assessed IgE production in affected individuals. These studies further suggest there is a need for more in-depth research to elucidate the AGS onset mechanisms. However, they introduced the concept of blood type as a factor in acquiring red meat allergy. On the other side, studies have reported genetic predisposition, or atopy, as a critical factor in food allergies (Commins et al., 2009). Individuals with atopy tend to exhibit heightened type I hypersensitivity in immune responses, with excessive IgE production against common allergens, such as mites, dander, and foods (Justiz et al., 2021). The increase in anti-α-gal IgE levels correlates with total IgE levels; thus, atopy was hypothesized to be an associated factor in AGS development (Fischer et al., 2017). One cross-sectional sero-prevalence study described a correlation between anti-α-gal IgE levels following tick bites and atopy (Gonzalez-Quintela et al., 2014). In contrast, another study reported that there is no correlation between AGS and atopy (Commins et al., 2012). Since, atopy is linked with multiple genetic factors, age, ethnicity as well as environmental factors hence it is difficult to reject correlation with AGS. A broader study involving wider population considering all possible factors could decipher possible correlation of AGS and atopy.

Various studies have shown that equilibrium of microbiota in epithelial barrier is vital for protection against allergic sensitization and disease development (Iweala and Nagler, 2019; Ohshima, 2013). Studies have demonstrated that shifting of a usual diet towards high fat, low fiber, highly processed food, and indiscriminate use of antibiotics can cause microbiome dysbiosis (Weissis and Hennet, 2017; Wypych and Marsland, 2018). Microbiome dysbiosis is linked to the rising number of cases and prevalence of food allergies in humans (Iweala and Nagler, 2019; Shreiner et al., 2008). Two central hypotheses could explain such phenomena: the first states that imbalanced microbiota and microbial stimulation can lead to a rise in food allergies. The second states that the imbalance of mucosal-barrier regulation could lead to oral tolerance loss. Current trend shows that incidence of AGS is higher in countries with higher number of allergic cases (Graham-Rowe, 2011; Loh and Tang, 2018; Cabezas-Cruz et al., 2019; Binder et al., 2021). This trend is explained by hygiene hypothesis (HH) which states that exposure of allergen or microbiome in environment at early stage in life reduce risk of development of allergies. Existing literature suggests that HH is linked with food allergy especially in children with atopy (Iweala and Nagler, 2019). Contrary to that, AGS has been reported mostly in humans with no history of atopy (Wilson and Platts-Mills, 2019; Binder et al., 2021). Interestingly, AGS is reported in people living in rural and urban setting across the globe (Cabezas-Cruz et al., 2019; Binder et al., 2021). Based on existing data it is not possible to reject or accept correlation of AGS with hygiene. There is not enough scientific evidence to accept or reject HH and more research is needed to decipher a link between AGS and HH.

Discovery of α-gal epitope in cat dander prompted researchers to investigate its association with AGS. Though, significant research efforts are made in this area, possible association of cat ownership, α-gal sensitization and AGS has not yet been fully rejected or established. There exist two contrasting research reports, one study reports increased level of anti-α-gal IgE, however, a study rejected this possibility because anti-α-gal IgE positivity was not observed when association was investigated by skin prick test (Gonzalez-Quintela et al., 2014; Bircher et al., 2017).

The jury is out on the question of why only a few tick species can induce AGS. Based on several studies, tick associated factors which can contribute towards AGS can be divided into two categories a) intrinsic factors and b) extrinsic factors. Tick intrinsic factors include the tick microbiome, tick glycosylation machinery, as well as host-seeking and feeding behaviors. On the other hand, tick extrinsic factors may include the geographical distribution of ticks and tick–predator interactions, which limits the tick population. Knowledge of distribution of ticks might be beneficial to evaluate health risk such as AGS, driven by expansion of tick populations. Since distribution of ticks is very wide and driven by multiple ecological factors, inclusion of such factors in ecological models to predict AGS risk assessment, rate of actual exposure and tick bites in certain areas must be considered. Role of tick’s intrinsic factors is vital in the context of AGS development. A tick attaches to the host by piercing the skin with its barbed mouthpart (the chelicerate). When anchored into the host skin, it continuously secrets saliva with a plethora of antigens (Francischetti et al., 2009). During blood meal, tick mouthparts induce trauma to the host skin through the breach of skin barrier integrity. This can lead to disruption of the host skin microbiota and facilitate the introduction of tick-borne microbiota (pathobionts) (Bonnet et al., 2017). Tick microbiome can contribute to AGS development possibly *via* sensitization process during tick bite or by increasing α-gal signature in the tick. Key details regarding role of tick microbiome are discussed in earlier section. There is a gap in our knowledge of the tick microbiome and its link to AGS. New cutting-edge tools to manipulate the tick’s microbiome are needed to understand the emergence of this unique allergy. A comparative analysis of the microbiome composition residing within different tick species, microbial profiles at tick bite sites may identify the microbial signature involved in AGS development. An urgent question is why, in contrast to other tick species, does one tick species decorates its saliva antigen with the α-gal epitope? Presumably, a tick’s robust glycosylation machinery is involved in the process of adding α-gal to saliva antigens which is responsible for AGS development. Our knowledge of the glycosylation machinery’s fitness in different Ixodid tick species is in its early stages. Our earlier work showed that there is a significant difference in the N-glycome profile of *A. americanum*, a tick linked with AGS in comparison to another hard tick, *A*. *maculatum*, which is not associated with AGS (Crispell et al., 2019). Furthermore, in the same study, results from the basophil activation test (BAT) demonstrated that tick’s ability to elicit α-gal sensitization is variable between species (Crispell et al., 2019). Indisputably, glycosylation is a conserved machinery in several taxa of eukaryotes; however, divergence is observed in the subsequent steps, which can generate interspecies- and intraspecies-specific N-glycan profiles (De Pourcq et al., 2010). Ginsberg et al., (2021) found a link between Lyme disease and the tick’s host-seeking behavior. The preferred host and latitudinal differences in tick host-seeking behaviors are associated with a specific tick-borne disease’s distribution in a particular geography. Since a tick’s host-seeking behavior varies among tick species, it can directly affect host encounter and incidence of certain diseases such as AGS. The expansion of the Lone Star tick population from its previously established territories into new geographic ranges has also been suggested as the cause of the increased numbers of AGS cases in new territories (Monzón et al., 2016; Raghavan et al., 2019). This increase in population and expansion into new areas may have been due to a surge in the deer population and the tick’s intrinsic ability to succeed in a diverse or changing environment by manipulating the expression of stress-mitigating molecules (Monzón et al., 2016; Raghavan et al., 2019; Bullard et al., 2019; Commins, 2020). Wilson et al., (2021) showed a negative correlation between AGS cases and fire ant invasion in the established tick population territories. Since fire ants are known tick predators, it is hypothesized that tick–predator interactions also affect AGS incidence in Lone Star tick endemic areas.

## Tick Bite, Host Response and Development of AGS

How a tick bite leads to host sensitization and AGS development is poorly understood. The tick–host interface is a complex battleground. When the tick disrupts the epithelial barrier by causing injury to the host skin by its barbed hypostome, a host driven hemostatic response initiates (Glatz et al., 2017). Hemostatis is the host’s innate defense mechanism which is activated against the mechanical injury and includes blood coagulation, platelet aggregation, and vasoconstriction (Francischetti et al., 2009; Kotál et al., 2015). In addition to that, during early stage of the tick’s attachment to the skin, humoral and cellular parts of host innate immune system respond with complement activation, inflammation and *via* infiltration of leukocyte to the bite site (Francischetti et al., 2009). Following the tick bite, activation of keratinocytes, endothelial cells and skin resident leukocytes occurs when they encounter tick saliva or hypostome (Wikel, 2018). Release of antimicrobial peptides, pro-inflammatory chemokines and cytokines including interleukin-8 (IL-8), interleukin-1β (IL-1β), tumor necrosis factor (TNF) by various leukocytes recruits various inflammatory cells including neutrophils (Wikel, 2018). Afterwards, adaptive immune system also branches out in which, activated T and B cells (in case of secondary infestation) increases the inflammatory response to tick *via* release of cytokines and production of antibodies targeted against tick to further activate complement as well as sensitize mast and basophil cells (Kotál et al., 2015; Wikel, 2018). To maintain uninterrupted blood uptake by evading host immune response, tick secretes complex mixture of molecules to reduce pain and itch to the host during feeding. These molecules include saliva vasodilators, inhibitors of platelet aggregation and molecules capable of inhibiting blood coagulation cascades (Francischetti, 2010; Mans, 2011; Kazimirová and Stibrániová, 2013). Furthermore, ticks also release various salivary molecules which are involved in lowering production of pro-inflammatory cytokines such as TNF-α, interlukin-12 (IL-12) as well as increasing production of anti-inflammatory mediators for example interleukin-10 (IL-10) and transforming growth factor beta (TGF-β) (Ferreira and Silva, 1999; Wikel, 2018). Following tick bite, skewing of T helper 1 (TH1) response towards T helper 2 (TH2) is vital in the process of AGS development. After distruption host skin epithelia by tick bite, in the process of wound healing, M2 macrophages are involved in suppression of inflammation by upregulating anti-inflammatory cytokines like IL-10 or TGF-β to alleviate an exaggerated TH1 cell response (Krzyszczyk et al., 2018). In addition, inhibitory action on pro-inflammatory cytokines (such as IL-1) by salivary molecules further promotes action of M2 polarized macrophages, which leads to inhibition of TH1 immune response and shifts host immune response towards TH2 (Wikel, 2018). Additionally, various other components of tick saliva such as prostaglandins, sphingomyelinase, and a cysteine protease inhibitor are reported to be vital in shaping the innate immune response by inducing TH2 profile (Alarcon-Chaidez et al., 2009; Oliveira et al., 2011; Carvalho-Costa et al., 2015; Lieskovská et al., 2015). Another study also reports that shifting of host immune response towards TH2 leads to stimulation of the humoral immune response and promotes B cell proliferation and induction of antibody production (Berger, 2000). Various studies report that repeated infestation of mice with ticks increases the level of TGF-β and leads to gradual increase in level of IL-10, IL-4 as well as increased TH2 response (Alarcon-Chaidez et al., 2009, Ferreira and Silva, 1999). During tick feeding, differential expression of salivary molecules which are capable of reducing pro-infmammatory cytokines such as IL-12, IL-1 β or TNF-α as well as production of anti-infmammatory mediators i.e IL-10. All of these events mentioned earlier further contributes towards maintenance of skewed TH2 immune response and contribute to AGS development (Ferreira and Silva, 1999).

**Figure 2:**
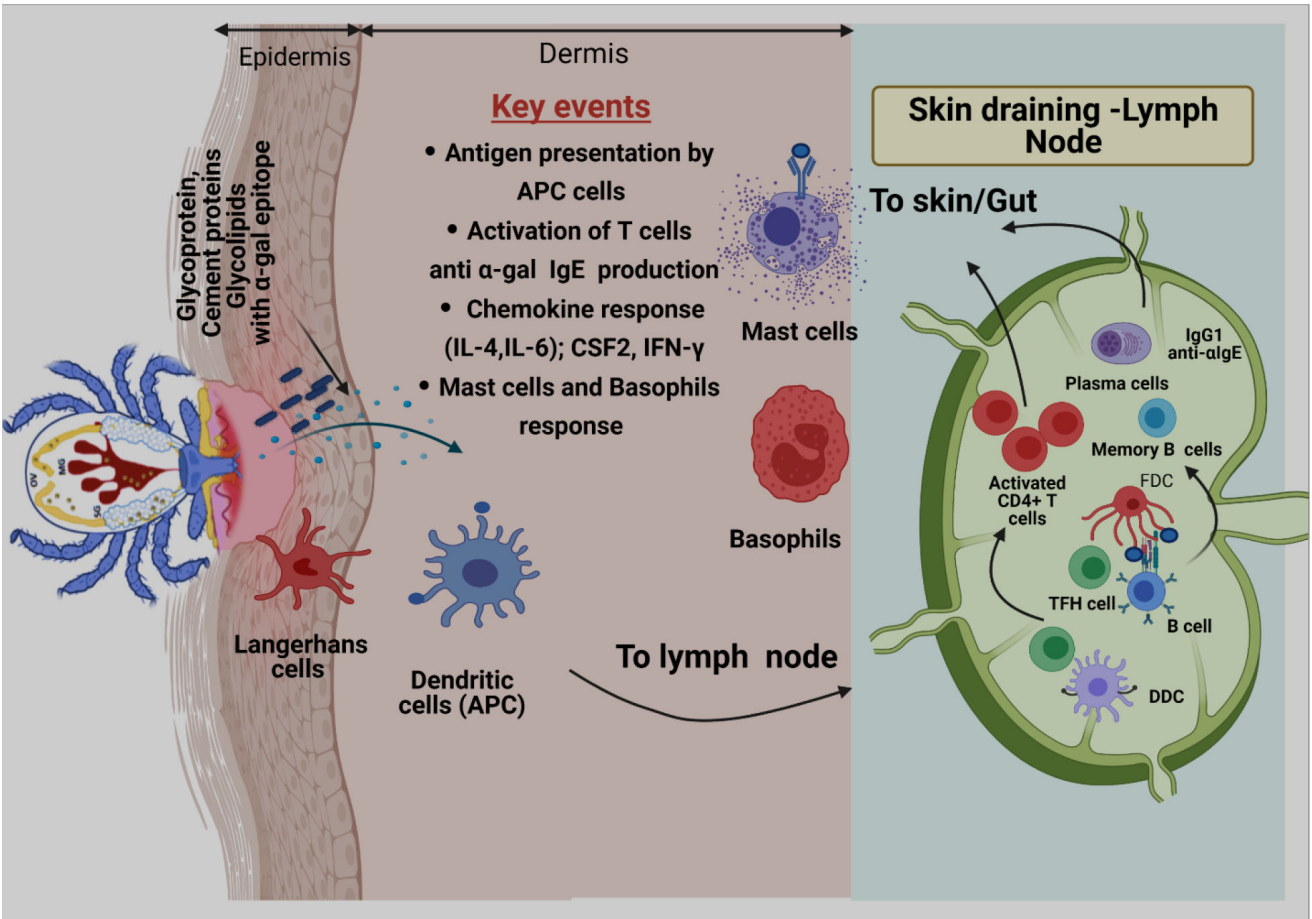
Proposed model of α-gal sensitization from tick bites. Skin is comprised of three layers: epidermis, dermis, and hypodermis. Antigen-presenting cells (APCs), including Langerhans cells (LCs) and dermal Dendritic cells (DCs) residing in epidermis and dermis, respectively, respond to tick-secreted antigens, such as glycoproteins, glycolipids, and tick cement-containing α-gal moieties. After antigen exposure, APCs process antigen, migrate to skin-draining lymph nodes, and participate in allergen sensitization. During this process, naïve T cells are primed through presentation of tick α-gal antigens by LCs and dermal DCs within skin-draining lymph nodes. Activated CD4^+^ T cells subsequently traffic to the skin through blood and lymphatic vessels. Cognate T cell help, provided by T follicular helper (TFH) cells, to α-gal-specific B cells leads to germinal center responses, positive clonal selection of B cells *via* recognition of native antigens retained by follicular dendritic cells (FDCs), and the development of memory B cells and plasma cells. After clonal selection, B cells migrate to the tick bite site on the skin to manifest allergic responses by presenting antigens to T cells, secreting proinflammatory cytokines, and secreting α-gal-specific antibodies (anti-α-IgE) that ultimately triggers activation of mast cells and basophils and allergic response.

Review including key details related to tick bite development of B cells in context AGS can be found elsewhere (Chandrasekhar et al., 2020). Briefly, the initial encounter of allergen with host immune cells happens at the skin epithelium during the tick bite. It is reported that tick saliva contains high concentration of Prostaglandin E2 (PGE_2_), which is found to be involved in reduction of inflammation and recruitment of macrophages. Hence, these events help further to create a suitable environment to drive immune response towards TH2 profile (Williams, 1979; Poole et al., 2013). Additionally, research has shown that PGE_2_ can directly induce class switching of the specific B cells to produce IgE (Gao et al., 2016). The development of B cell-producing antigen-specific IgE Abs is a hallmark of allergic responses following antigen exposure. Cabezas-Cruz and Valdes (2014) reported that tick saliva induces responses like a venom antigen, which not only counteracts with the immune system but also drives immune sensitization. Initial encounter between tick-secreted saliva antigen and host immune cells happens at the skin epithelium during a tick bite. Antigen presenting cells (APCs) present in skin more specifically, Langerhans cells (LCs) and dendritic cells (DCs) recognize, capture, and process salivary α-gal antigens and migrate to skin-draining lymph nodes to participate in sensitization of B cells (**Figure 2**) (Chandrasekhar et al., 2020). After clonal selection, sensitized B cells migrate to the tick bite site in the skin to manifest allergic responses by presenting the antigen to T cells, secreting proinflammatory cytokines, and α-gal-specific antibodies that eventually trigger mast and basophil cell activation (Chandrasekhar et al., 2020).

During AGS development and allergic response various human cells are involved. In early sensitization stage skin resident antigen presenting cells are vital. Skin is compartmentalized into two layers i.e., epidermis and dermis by basement membrane. In these layers specialized antigen presenting cells (APCs) namely Langerhans cells, a subpopulation of Dendritic cells (DC) are present. DCs play central role in connecting both innate and adaptive immune systems. Especially in context of tick bite and sensitization these cells are involved in internalization and processing of α-gal bound antigens injected by tick while feeding (Kashem et al., 2017). Since, DCs do not produce cytokines required for TH2 cell differentiation for the development of AGS, tick salivary component like PGE2 are required to polarize DCs towards TH2 (Carvalho-Costa et al., 2015). After completion of sensitization, mast cells play central role in allergic response. Mast cells are localized in tissue and express IgE binding receptors (FcεRI). During activation stage cross linking of FcεRI bound IgE Abs occurs that leads to degranulation of mast cells to release allergy specific mediators along with TH2 cytokines (i.e., IL-3, IL-4) (Mcleod et al., 2015).

Basophils are important circulating granulocytes, which are involved in chronic allergic responses as well as tick acquired resistance in non-natural hosts (Sokol et al., 2009; Karasuyama et al., 2020). Like mast cells basophil cells also express FcεRI receptors to bind IgE and release histamine and related mediators after activation and degranulation (Sokol et al., 2009). In addition, that study suggests basophils might be involved in antigen presentation and initiation of TH2 immune response and cytokine production (Sokol et al., 2009).

## Conclusions

AGS is a newly emerged food allergy reported in different parts of the world and is associated with tick bites. The α-gal epitope (galactose-α-1,3-galactose), an oligosaccharide, is the prime culprit responsible for AGS. The exact mechanism of how a tick bite causes human sensitization against α-gal and leads to the development of AGS, is poorly understood. Identification and functional characterization of tick-associated molecules are vital for developing interventions to prevent and control this disease. The presence of the α-gal epitope in tick species has been confirmed. However, mechanism of synthesis, origin and delivery of these molecules at the tick–host interface are subject of investigation. Furthermore, there is a gap in our understanding of how tick microbiome contributes towards AGS development. Comparative analysis of the microbiomes maintained by the ticks along with their genetic machinery using genomic and transcriptomic approaches may reveal the genes contributing to α-gal synthesis. Future research should be focused on 1) identifying and characterizing key tick salivary molecules decorated with the α-gal epitope, 2) the molecular mechanism of α-gal synthesis, 3) the mechanism of α-gal delivery in tick–host interaction interface, 4) the process of sensitization against α-gal during tick hematophagy and involved immune pathways, and 5) the role of various host-associated and tick-associated factors contributing to the development of AGS.

## Author Contributions

SRS searched the literature and wrote the initial draft of manuscript. SK searched the literature and wrote the manuscript. All authors contributed to the article and approved the submitted version.

## Funding

This work was supported by USDA National Institute of Food and Agriculture awards, 2017-67017-26171 and 2016-67030-24576; the National Institutes of Allergy and Infectious Diseases award, RO1 AI135049; and the National Institutes of General Medical Sciences award, P20RR016476.

## Conflict of Interest

The authors declare that the research was conducted in the absence of any commercial or financial relationships that could be construed as a potential conflict of interest.

## Supplementary Material

Supplementary Figure 1 Distribution of tick species known to be associated with alpha-gal syndrome.

Supplementary Figure 2 Summary of Alpha-gal Sensitization and associated symptoms.
