# Supplementary figures and images for: Tick Saliva and the Alpha-Gal Syndrome: Finding a Needle in a Haystack
Source: Front Cell Infect Microbiol. 2021 Jul 20;11:680264. doi: 10.3389/fcimb.2021.680264 (PMC8331069; doi:10.3389/fcimb.2021.680264)

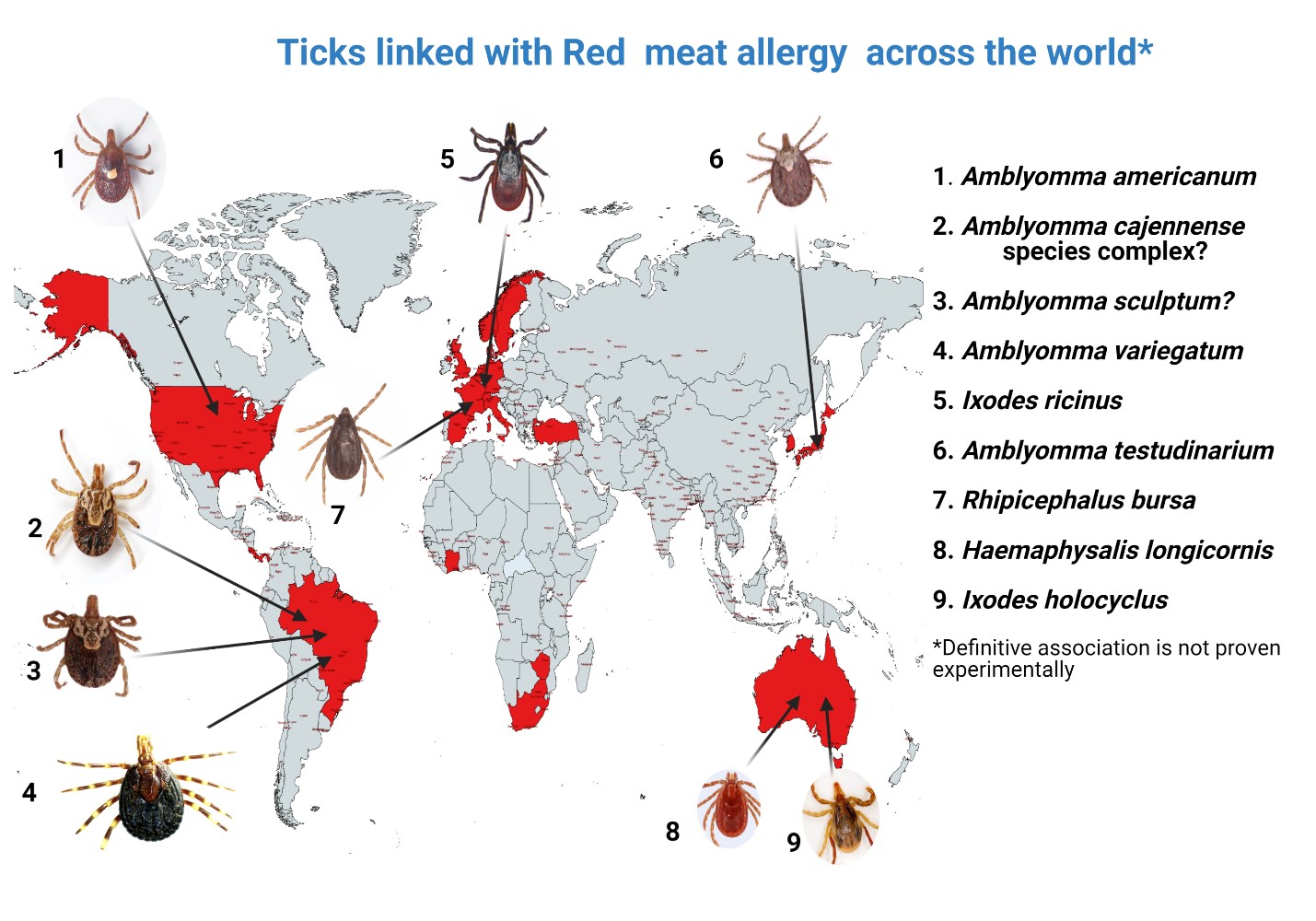

Supplement: Supplementary Figure 1 — Distribution of tick species known to be associated with alpha-gal syndrome. [file Image_1.jpg]

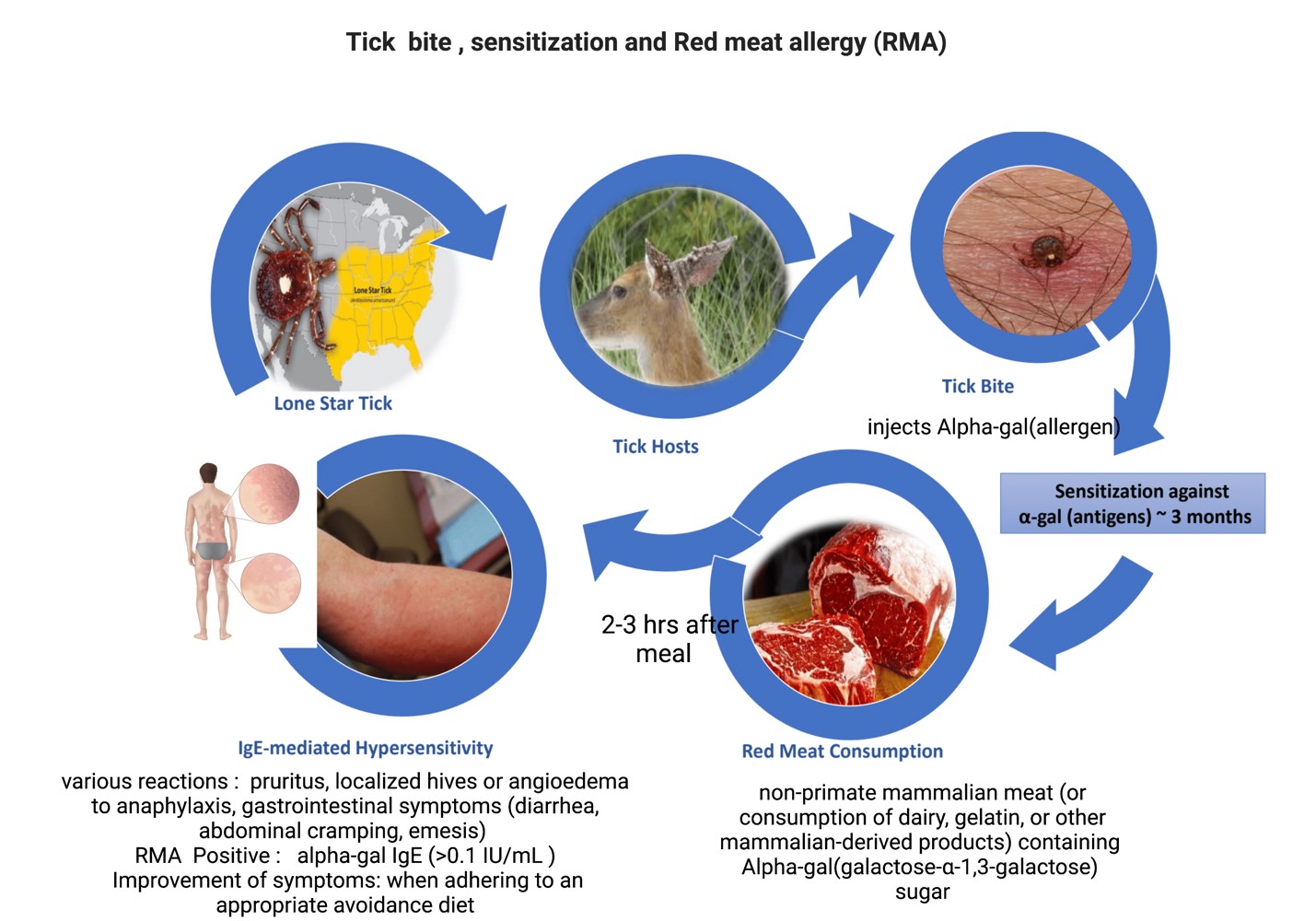

Supplement: Supplementary Figure 2 — Summary of Alpha-gal Sensitization and associated symptoms. [file Image_2.jpg]
